# Developing a Scale for Measuring the Fundamental Movement Skills of Preschool Children in China

**DOI:** 10.3390/ijerph192114257

**Published:** 2022-11-01

**Authors:** Yong Chen, Ying Gu, Ying Tian, Hyunshik Kim, Jiameng Ma, Xuefeng Jia, Lianlian Qin

**Affiliations:** 1 Department of Physical Education, Huaiyin Normal University, Huai’an 223300, China; 2 College of Sports Science, Shenyang Normal University, Shenyang 110034, China; 3 Faculty of Sports Science, Sendai University, Shibata 9891693, Japan; 4 School of Physical Education, Shenyang Sports University, Shenyang 110102, China; 5 Spring Kindergarten, Shenbei New District, Shenyang 110034, China

**Keywords:** preschool children, fundamental movement skills, measurement tool

## Abstract

Children aged 3–6 years (preschool children hereafter) are in a critical and sensitive period of developing fundamental movement skills (FMS). While appropriate measurement tools can accurately evaluate their FMS development, such a scale is lacking in China. In this study, a suitable scale for measuring the FMS of preschool children in China was developed by combining process- and results-oriented evaluation methods. The subjects of this cross-sectional study included 21 experts and 358 preschool children (188 boys and 170 girls). Based on a literature review, items suitable for measuring the FMS of preschool children in China were selected, and the final measurement scale includes 12 items in three dimensions: physical locomotion skills, object control skills, and physical stability skills. After a battery of tests to assess its suitability, including inter-rater reliability, test/retest reliability, homogeneity, and construct validity, we find that the proposed measurement scale has good reliability, validity, and sensitivity. This scale reflects the development level of the FMS of preschool children in China and can be used to monitor the FMS of this population in the future.

## 1. Introduction

Movement skills are essential in ensuring survival and promoting physical development through sports [1]. As most movement skills are acquired in early childhood, their development forms the basis for young children’s physical growth as well as behavioral activities such as learning [2]. Hence, they are an essential guarantee for human survival as well as reproduction, adaptation, and transformation of the objective world [3]. Moreover, the correct development of young children’s motor competence can promote physical activity in the future [4]. Therefore, the movement skills learned in this development stage are termed “fundamental movement skills,” or FMS hereafter [5]. FMS, which are non-naturally occurring abilities, can be divided into physical locomotion skills, object control skills, and physical stability skills according to the movement function [6].

Studies have found that good FMS can improve children’s confidence in future sports participation [7,8], and will also lay a good foundation for them to learn complex sports skills in the future [1]. Foweather et al. believe that the development of FMS is crucial to mastering various sports, competitions and dances [9]. However, the lack of appropriate environmental conditions during the learning period of fundamental movements and the lack of proper development of fundamental movements is the reason for the weak movement ability in adulthood [10]. FMS not only plays an important role in children’s aca-demic performance [11] and social cognition [12]. Movement can provide children with cognitive experience, enrich cognitive objects, and enable children to have more opportunities to identify the essential characteristics of things from their external manifestations, and then gain an understanding of the nature of things [13]. However, due to the immaturity of motor development in early childhood, individual cognition mainly comes from perceptual ability [14]. The development of movement skills also affects children’s social adaptability [15]. The key to the interaction between children’s social development and movement lies in the evolution of children’s cognition of self-ability [16]. In the learning atmosphere of various movement skills children can get a positive emotional experience and help them gain self-confidence and success; in the process of movement skill acquisition, help children understand social rules, acquire good social behaviors, and reduce aggressive behaviors [8]. Therefore, the good development of FMS will have an important impact on the physical and mental health of children [17].

FMS development is central to early physical activity [18]. Children gradually master these fundamental abilities through organized education and learning. As these movements involve all body parts, FMS is also the basis for developing sports skills [19,20,21,22]. As noted above, FMS usually develops in early childhood [23,24,25]. Children aged 3–6 years (preschool children hereafter) [26] are in a critical and sensitive period of FMS development [27]. Early childhood FMS development not only affects the learning and development of movement skills after individual growth but also promotes many aspects of one’s own physique, cognition, perception, nervous system development, creative thinking, understanding, self-expression and social ability [28,29,30]. Many FMS are formed before the age of 8 [31]. The development of FMS can correspondingly improve children’s self-confidence and motor proficiency [32]. On the contrary, if the FMS is not recognized and remedied at the right time, Children may have lifelong problems in movement development [33]. Additionally, poor movement ability is usually accompanied by physical weakness [34], with the risk of being overweight and obese [35]. Three to six years old is a critical period for children to learn FMS. This is a critical period for developing children’s sports psychology, sports interest, habits, cognition and comprehensive sports quality. Suppose they receive scientific, systematic and timely training during this period. In that case, children’s sports psychology, sports interest, sports habits, sports cognition and comprehensive sports quality will be optimally developed, and they will also help children develop good sports habits for a lifetime, to benefit young children for life [36]. Therefore, missing this period of FMS development can lead to children’s inactivity, uncoordinated movements, lack of sports confidence, and other problems in the future [12,37]. Thus, proficiency in FMS contributes to children’s physical, cognitive, and social development [38], laying a foundation for forming various movement skills in the future and cultivating lifelong physical exercise habits [39].

In the past 10 years, obesity and physical decline in Chinese adolescents and children have become significant problems that plague the education and sports administrative departments [40]. Insufficient physical activity and static lifestyles in Chinese children and adolescents have become common [41]. In the teaching practice, games are the main content, and the training of motor skills is ignored, which is also the essential reason for “obesity”, “low physical fitness”, “incoordination of movement”, “poor resistance” and “cowardly character” in preschool children in our country. The problem arises [42]. Studies have shown that the development of FMS in Chinese preschool children is still at a low level [43], and there is no assessment tool for fundamental skills based on my country’s national conditions [44]. In September 2019, the Outline for Building a Powerful Country in Sports [45] proposed, “Promote the development of children’s sports, improve the policy and security system, promote the construction of children’s sports projects and children’s sports equipment standard system, and guide the establishment of children’s sports curriculum system and teacher training system”, indicating the importance of the work of early childhood sports. In June 2018, Beijing Sports University officially released the first domestic Guidance manual of Sports Guide for Preschool Children (3–6 years old) (expert consensus version) [46]. The emergence of the “Guide” will further refine the direction and method of children’s physical education and have a more targeted guiding significance for kindergartens and families. In the same year, in August 2018, at the kick-off meeting of the “China Qing-Ling Song Foundation Plei Children and Youth Physical Education and Healthy Development Special Fund”, Kaizen Wang (Vice Chairman of the National Early Childhood Sports Development Committee) published “The ‘hot’ and ‘Need’” keynote speech, clearly pointed out that my country currently lacks children’s special sports skills learning (practice) standards, so there is an urgent need to research children’s sports-related systems and standards [47]. A series of policy documents such as the “Healthy China 2030” Planning Outline [48] and the Youth Sports Activity Promotion Plan [49] have proposed that young people should basically achieve proficiency in 1 or 2 sports skills. On this basis, Wang Jun et al. believe that FMS is the basis of advanced motor skills with sports as the carrier, and the above policy documents are the requirements for forcing children to develop FMS [17].

FMS measurement is widely used in developed countries to determine the growth and development of children and adolescents [50,51]. Globally, scholars have already started to study related aspects [52], and movement ability is being introduced into the field of child health promotion by sports epidemiologists. The establishment of China’s preschool FMS evaluation system is still in its infancy, but FMS has gradually become a new field in the development of the movement of Chinese children [17]. However, at present, the assessment targets in some developed countries are mainly to screen for developmental delays and developmental disorders or to assess the overall development level of children. Theoretical structures also vary. Moreover, most of these commonly used evaluation tools are developed with the Western population as the norm. Due to the objective existence of cultural and ethnic differences between the East and the West, it is doubtful whether these evaluation scales can be directly applied to the physical measurement of Asian children. However, methods and standards for evaluating the FMS of preschool children are lacking in China. Hence, there is a knowledge gap between Chinese and foreign children in this critical period of learning movement skills [40]. Therefore, Chinese researchers have proposed that it is necessary to strengthen com-prehensive multidisciplinary research and develop measurement tools and evaluation standards that align with Chinese children [42,53]. The development of an assessment tool with Chinese children as a data model should be put on the agenda to evaluate the level of FMS in Chinese children [44]. Xin Fei et al. proposed to develop an FMS evaluation tool suitable for Chinese children based on the use of foreign FMS evaluation tools (such as TGMD-2) [54]. On this basis, it is particularly important to develop an evaluation system that conforms to the FMS development of preschool children in my country and to establish a development evaluation model, so as to provide a direct and indirect way for preschool children to achieve lifelong awareness of physical activity.

The current mainstream evaluation methods for children’s movement skills development are developed by scholars in the United States, Germany and other countries based on the data of their children and adolescents’ movement skills, focusing on the application of norm-referenced evaluation in evaluation. However, most assessment tools have the disadvantage that their validity is weakened by cross-cultural assessment. Its data norm cannot be accurately applied to Chinese children [44]. Chinese scholars Jing Li et al. [43] used TGMD-2 and TGMD-3 to test Chinese children and adolescents, they found that the two items of hitting the fixed ball and the underhand toss were derived from the actions of American students hitting a baseball. These two movements have low scores on the test of Chinese children, which are more difficult for Chinese children and are not very suitable for Chinese children [55]. Researchers believe that FMS is divided into three dimensions: physical locomotion skills, object control skills, and physical stability skills [6], while TGMD lacks the evaluation of this dimension of physical stability skills. We hope that through our continuous exploration, with Chinese children as the data norm, compile measurement tools and evaluation standards that are in line with Chinese children. In the selection of test indicators, it is possible to establish an indicator system that can comprehensively and objectively reflect children’s movement development, and develop FMS assessment tools suitable for young children in China.

The *National Physical Fitness Measurement Standards (Young Children)* [56] formulated by the General Administration of Sport in China in 2003 provide body shape and quality indicators, while the content mainly uses results orientation as the evaluation method. In 2012, the Ministry of Education issued its *Guidelines for the Learning and Development of Children Aged 3–6* [57], which proposed developing movement (i.e., children’s physical strength, endurance, balance, sensitivity, coordination, and other activities) as a goal of early childhood health outcomes [58]. However, again, the results orientation of this guide cannot comprehensively evaluate these abilities. By contrast, due to the FMS teaching-oriented educational thought, western countries mainly focus on the process-oriented evaluation standards in the evaluation of children’s physical examination. The advantage of this evaluation standard is that it can test the quality of children’s action performance, and can more accurately evaluate the coordination, balance, and sensitivity of children’s movement process [54]. This provides a good reference value for our early childhood educators to evaluate these indicators in the future.

Therefore, based on the preceding sections, this study develops a scale to measure the development of the FMS of Chinese preschool children that combines a process and a results orientation [44,57]. It contributes to the literature by enriching the existing theories of young children’s physical development in China. Its scale could help strengthen children’s physiques, promote the development of their bodies and minds, and contribute to helping them develop good exercise habits.

## 2. Materials and Methods

### 2.1. Study Participants

A convenience sample from northeastern Chinese that volunteered to participate in the “Study on the Improvement of Life Habits among Children in East Asia” was selected in consideration of the study’s purpose [59]. In this study, 21 experts and 358 preschool children (188 boys and 170 girls) were selected in China, among whom 60 preschool children (30 boys and 30 girls) participated in the testing of the preliminary measurement scale and 298 preschool children (158 boys and 140 girls) participated in testing the final measurement scale, as illustrated in Table 1. The 21 invited experts included those who investigate young children’s movement skills and psychological development. These experts comprised 13 university teachers (five doctoral supervisors and eight master’s supervisors), six kindergarten principals, and two movement development researchers from the Institute of Child Care. They all had more than 12 years of working experience in movement skills and early childhood education.

The participants were randomly selected from preschool children in five-star kindergartens in northeast China. Informed consent was obtained from and signed by the kindergartens and parents before the test. The study was approved in advance by the Ethics Committee of the Sendai University School of Sports Science (Approval No.: SU29-22). Children’s ages were calculated following the *National Physical Fitness Standards Manual (for Young Children)* [56].

### 2.2. Study Procedure

**Figure 1:**
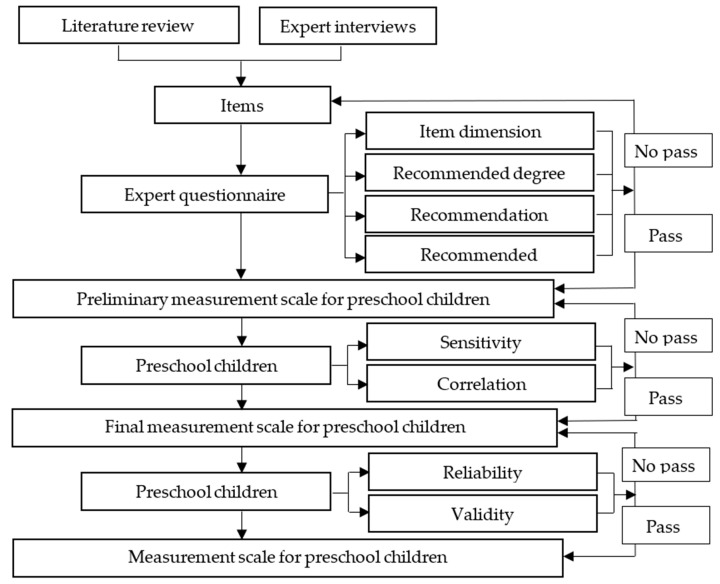
Study procedure.

The study Procedure is shown in Figure 1.

First of all, a large number of existing FMS test scales and guidelines, as well as academic research and books related to the development of children’s FMS, are sorted out. Through interviews, symposiums and questionnaires with parents, kindergarten directors and teachers, we learned about the physical and motor development of preschool children, as well as their sports habits, and analyzed the problems in the evaluation system of basic motor skills, such as incomplete evaluation objectives, unreasonable measurement tools, and inappropriate test contents. After summing up the results of the two, we will sift out the evaluation items that do not apply to children aged 3–6 in China due to age, project description, and project scoring standards, experts suggest, China carries out fewer sports and other reasons, and establish a project pool.

Secondly, the Delphi method is used to invite experts and Issue questionnaires to experts for comments. Through data statistics, survey experts’ consensus on the three dimensions, recommendation degree, recommendation divergence and recommendation concentration of each project in the corresponding dimensions. To form the preliminary content of the measurement scale of the FMS evaluation indicators for preschool children.

Thirdly, 60 children aged 3–6 years were measured with the preliminary measurement scale to check the discrimination of the draft scale and the correlation between the items and their dimensions. Unqualified items were removed from the draft project pool to form the final measurement scale.

Finally, 298 preschool children aged 3–6 years were tested with the final measurement scale to check the interrater reliability, retest reliability, homogeneity reliability and construct validity, and the research on FMS evaluation methods for preschool children aged 3–6 years was completed.

#### 2.2.1. Determination of the Items of the Measurement Scale

A lot of literature retrieval work has been carried out in the early stage. First of all, used Web of Science, EBSCO, Science Direct, CNKI, Wanfang, VIP database (China Science and Technology Journal Database) and other search platforms to search for English and Chinese keywords “fundamental movement skills”, “FMS”, “motor skills”, “preschool”, “young children”, “child”, “preschool”, “children’s movement skills” and “children’s fundamental movement skills”. The widely used children’s movement development scale and guide, the relevant academic research on children’s movement development included skills testing tools, classification and definition of children’s basic movement skills, characteristics of children’s movement development and intervention of children’s movement skills were retrieved. Secondly, more than 50 books on children’s motor skills were read. Collect and sort out the retrieved documents and books. Although the original intention, content form, and testing object of these evaluation tools are different, they are all applied to the evaluation of FMS by scholars [56] (Table 2).

#### 2.2.2. Method of Evaluating the Measurement Scale

A results-oriented evaluation is an evaluation carried out after an activity, with the purpose of assessing and identifying the results of the activity [69,70]. Process-oriented evaluation, or formative evaluation, is an evaluation conducted in the middle of an activity [69]. Result-oriented evaluation and process-oriented evaluation come from the formative evaluation and summative evaluation in The Methodology of Evaluation written by Harvard University evaluation expert Michael Scriven in 1967 [71]. The result-oriented evaluation focuses on the “judgment” of results [71]. In contrast, the formative evaluation does not aim at distinguishing the goodness of the evaluation objects, and does not pay attention to the classification of evaluation objects, but pays more attention to how to “improve” [71,72]. Process-oriented evaluation tends to the value orientation of “process” and “development”, which is the conceptual basis of process evaluation [73]. In foreign children’s FMS measurement and assessment, most evaluation methods are based on the process orientation of the quality of children’s movement performance. The test content of “National Physical Fitness Measurement Standard (Young children)” [56], which is a movement performance assessment for young children in my country, focuses on result evaluation to highlight the importance of children’s physical fitness. This result-oriented evaluation standard is simple to operate and intuitive. It reflects the physical health level but lacks the procedural evaluation of the motor development level in early childhood [74]. The evaluation method of this study measures both children’s movement processes and results, thereby offering a comprehensive scale for measuring the FMS development of preschool children by combining a process and results orientation.

**Figure 2:**
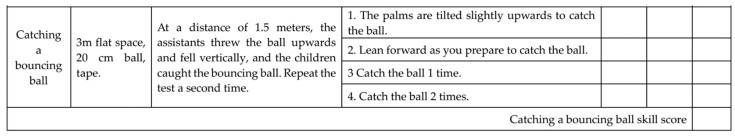
Catching a bouncing ball test score table.

For example, there are 4 skill standards for catching a bouncing ball. (1) The palms are tilted slightly upwards to catch the ball; (2) Lean forward as you prepare to catch the ball; (3) Catch the ball 1 time; (4) Catch the ball 2 time. A score of “1” is awarded for each standard that is met, and a score of “0” for failing to meet the standard (Figure 2).

#### 2.2.3. Delphi Method

To build the expert questionnaire, we referred to a large number of FMS measurement scales for young children globally [56,60,61,62,63,64,65,66,67]. Then, according to the requirements of *Theory and Application of Scale Preparation* [74], we deleted those unsuitable for preschool children in China (i.e., based on age, item description, item scoring standard, advice from experts, few exercises in China). After summarizing and sorting, we included 32 items comprising 13 for physical locomotion skills, ten for object manipulation skills, and nine for body stabilization skills. The 21 experts answered the questionnaire using a five-point Likert scale, ranging from “not recommended” (coded 1) to “highly recommended” (coded 5). After completing the questionnaire, they were invited to rate its quality and provide opinions to help build the preliminary measurement scale. Kendall’ W^a^ was used to test the consistency of expert opinions.

#### 2.2.4. Building the Measurement Scale

Selected preschool children (three to five times the number of items in the preliminary measurement scale recommended by the experts) were recruited to test the suitability of the preliminary measurement scale. By testing the correlations between the items and their dimensions, those items that did not meet the requirements were eliminated, leaving the final measurement scale. Selected preschool children (ten times the number of items in the final measurement scale) were recruited to test the suitability, inter-rater reliability, test/retest reliability, homogeneity and construct validity of the final measurement scale.

### 2.3. Statistical Analysis

The IBM SPSS Statistics for Windows software (2017, V 25.0, IBM Corp., Armonk, NY, USA) was used to analyze all the data. The questionnaire data and experts’ opinions were analyzed. In Delphi method, Kendall’s is used to check the consistency of experts’ opinions. The average score, standard deviation, coefficient of variation, cumulative percentage and other methods are used to investigate the recommendation degree, recommendation divergence and recommendation concentration of experts on each item in the corresponding dimensions. *p* < 0.05 was deemed significant. The average score was more than 4, the coefficient of variation (CoV) was less than 0.25, and the cumulative percentage (i.e., the proportion of scores of 4 and 5) was more than 70%.

The average score, standard deviation, CoV, and Spearman correlation test were then used to analyze the test results of the preliminary measurement scale. Those items with high sensitivity that were significantly related were selected (*p* < 0.01 indicated statistical significance). To distinguish subjects with different levels, the evaluation criteria of items needed high sensitivity; hence, the CoV of subjects’ scores should not be too low. According to the previous screening criteria for scale items [75,76], items with a CoV of less than 20% indicated poor discrimination and low sensitivity.

The Spearman correlation coefficient was used to evaluate the final measurement scale’s inter-rater reliability and test/retest reliability. The Cronbach’s α coefficient was used to test the homogeneity of the scale. Kaiser–Meyer–Olkin (KMO) and Bartlett’s spherical tests were used to test the scale’s construct validity. Here, *p* < 0.05 was again deemed significant.

## 3. Results

### 3.1. Determination of the Preliminary Content of the Measurement Scale

The preliminary measurement scale contained 32 FMS items in line with the physical development of preschool children in China. It included physical locomotion skills (13 items): straight line running, shuttle running, changeover running, one-foot jump, standing long jump, front slide, sidestep slide, running jump, jumping continuously with both feet sideways, stride jump, jumping jacks, synchronized jump on the same side, and jumping obstacle; object control skills (ten items): bouncing a ball in place, catching a ball with both hands, kicking a ball, throwing a ball up with both hands, hitting a ball with one hand over the shoulder, bocce ball, overhand pitching, catching a bouncing ball, holding a ball and walking, and horizontal racket hitting fixed table tennis; and physical stability skills (9 items): standing on one foot, hanging on a horizontal bar, walking on a balance beam, straight walking, standing back and forth on two feet, squatting, backward straight walking, forward rolling, and tiptoe walking.

To analyze the consistency of the 21 experts’ scores on the questionnaire, Kendall’s W^a^ analysis [77] was carried out on the collected data (Table 3), indicating that the opinions of the experts were unified. Hence, the collected questionnaire data could be used for subsequent analysis and processing.

### 3.2. Formulation of the Measurement Scale Items

Table 4 illustrates the questionnaire results for all 32 items. According to the analysis results and statistical requirements [78], those items with an average score of more than 4, a CoV of less than 0.25, and a cumulative percentage of more than 70% were selected to test the preliminary content of the measurement scale.

Based on the criteria above, Table 4 demonstrates that, of the physical locomotion skills, straight line running, shuttle running, one-foot jump, standing long jump, sidestep slide, and stride jump (six items) were chosen. Of the object control skills, bouncing a ball in place, catching a ball with both hands, kicking a ball, hitting a ball over the shoulder with one hand, and catching a bouncing ball (five items) were chosen. Of the physical stability skills, standing on one foot and walking on a balance beam (two items) were chosen.

To test the suitability of the 13 items of the preliminary measurement scale, preschool children that number 3–5 times the items of the scale should be selected for the test. Therefore, 60 preschool children were selected to test the preliminary measurement scale. The children’s performance in the test was scored, and items with certain usage situations, unclear descriptions, language differences, and unsatisfactory data were modified to form a measurement scale that can accurately and quickly measure the FMS development level of preschool children.

Table 5 illustrates the results of testing the preliminary measurement scale. First, since the measurement scale needs a reasonable degree of discrimination to distinguish subjects with different levels, the measurement items must be highly sensitive (i.e., the CoV of subjects’ scores should not be too low). Therefore, as noted above, we excluded items with a CoV of less than 20%, owing to their poor discrimination and low sensitivity. Among the 13 items in the preliminary measurement scale, shuttle running’s CoV was less than 20%, so it could not be included in the final measurement scale. Hence, the final measurement scale comprised 12 items. The items in the physical locomotion skills included straight line running, stride jump, one-foot jump, sidestep slide, and standing long jump. The items in the object control skills included bouncing a ball in place, catching a ball with both hands, kicking a ball, hitting a ball with one hand over the shoulder, and catching a bouncing ball. The items in the physical stability skills included standing on one foot and walking on a balance beam.

### 3.3. Testing the Final Measurement Scale

To test the final measurement scale, preschool children that number more than 20 times the 12 test items should be selected (i.e., more than 260 children); hence, 300 preschool children were randomly selected for this test. Two children did not complete the test for physical reasons. The test results were used to examine inter-rater reliability, test/retest reliability, homogeneity, and construct validity (Table 6).

#### 3.3.1. Testing the Inter-Rater Reliability of the Final Measurement Scale

Two teachers scored the 298 preschool children for all the test items, and a Spearman correlation test was used to analyze the scoring data. The results demonstrated that the highest correlation coefficient was 0.975, and the lowest was 0.844. All the items were significant at the 1% level, indicating that the different raters provided a unified implementation of the test content and method standards.

#### 3.3.2. Testing the Test/Retest Reliability of the Final Measurement Scale

Four weeks after the completion of the final measurement scale test, 36 children (more than 10% of the sample) of different ages were selected from the original 298 preschool children for the retest (six boys and six girls each aged 36–47 months, 48–59 months, and 60–72 months). The Spearman correlation coefficients of all the items were at least 0.7. The highest was 0.956, and the lowest was 0.835, and all the items had a significant correlation at the 1% level. The results indicated that the final measurement scale had high test/retest reliability, the two measurements of individuals were similar, and the evaluation method and standard had high stability.

#### 3.3.3. Testing the Homogeneity of the Final Measurement Scale

The Cronbach’s α coefficient was used to test the homogeneity of the scale. A Cronbach’s α coefficient of more than 0.7 indicates adequate reliability, and one ranging from 0.7 to 0.8 means high reliability. In this study, the lowest Cronbach’s α coefficient was 0.812, and the highest was 0.854, which were both above 0.8. This indicated that the final measurement scale had homogeneity. That is, its internal consistency was very good.

#### 3.3.4. Testing the Validity of the Final Measurement Scale

The KMO and Bartlett’s spherical tests were used to evaluate the results of the final measurement scale. Principal component analysis was used to extract the factors with eigenvalues above 1. The maximum variance orthogonal rotation method was then used to calculate the communality variance of each item in the scale and its factor loading.

**Figure 3:**
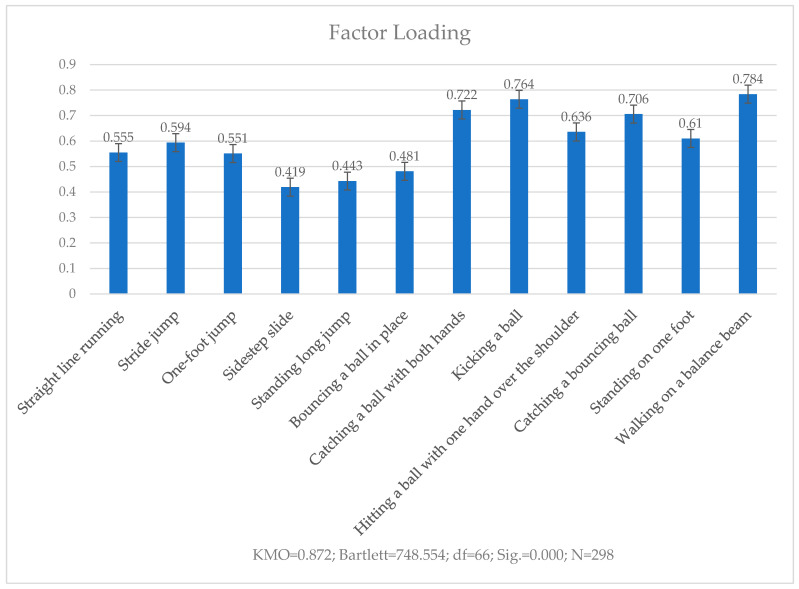
Factor loading of each item in the final measurement scale.

Figure 3 illustrates that KMO statistic was 0.872, above the threshold of 0.8, and that the significance value of Bartlett’s spherical test was 0.000 (*p* < 0.001), making a factor analysis suitable. the corresponding common factors of each item were all greater than 0.3, the minimum factor loading was 0.419, and the maximum factor loading was 0.784. Hence, the scale had good construct validity.

## 4. Discussion

Given the lack of an FMS measurement scale for preschool children in China, this study developed an appropriate 12-item scale for evaluating the development of Chinese preschool children’s FMS that combines a process-oriented and a results-oriented evaluation. This scale can comprehensively and objectively evaluate the FMS development of preschool children and lay a foundation for helping them develop good sports habits, form various movement skills, and increase their awareness of physical exercise. Compared with current measurement scales globally, our proposed scale presents several novelties, as discussed next.

### 4.1. Project Analysis

The FMS measuring scale in this paper has been described in a large number of measuring scales or guidelines, and the movements involved in different literature have been extracted in the compilation process. Straight line running has been described in TGMD-2, Guidelines for Learning and Development of Chinese Children aged 3–6 Years (2010), PGMQS, and National Physical Fitness Measurement Standards (Toddler section). One-foot jump, standing long jump, and stride jump have been involved in TGMD-2 and PGMQS. The movements of bouncing a ball in place, kicking a ball and catching a ball with both hands have been reported in TGMD-2, Guidelines for Learning and Develop-ment of Chinese Children aged 3–6 Years (2010), PGMQ, BOTMP-2, CDCC, etc. Walking on a balance beam and standing on one foot are involved in the Guidelines for Learning and Development of Chinese Children aged 3–6 Years (2010), M-ABC, PGMQS, BOTMP-2, CDCC and KTK.

### 4.2. Expert Analysis

The 21 experts recruited for this study recommended the questionnaire items based on their experience from several aspects, such as whether the item reflects the development level of young children’s movement skills, whether it is safe for young children in the test process, whether the test equipment is easy to operate, and whether the test scoring standard is simple and easy to understand. For example, the object control test involved throwing a ball up and catching a bouncing ball with both hands, and the throwing and dropping motions were simple. Focus was placed on the location and timing of the catch when the ball fell or bounced, but the direction of the throw was different; hence, experts believed that either item was desirable. Hitting a ball with one hand over the shoulder and overhand pitching was repetitive, so the experts suggested omitting either from the final measurement scale.

Likewise, several movements in the physical stability skills items were similar and related to balance. Therefore, the experts suggested removing front and back foot standing. Walking on a balance beam and straight-line walking were repetitive, and therefore, it was recommended to use only one. Moreover, tiptoe walking was also suggested to be removed. Shuttle running and changeover running were similar physical locomotion skills. Hanging on the horizontal bar and forward rolling seemed inappropriate for physical stability skills, so they were suggested to be removed.

### 4.3. Difference Analysis with Existing FMS Scales

#### 4.3.1. Evaluation Method

Chinese children’s physical fitness tests are currently based on the results-oriented *National Physical Fitness Standards (Young Children)* formulated by the General Administration of Sport in China in 2003 and the *Guidelines for the Learning and Development of Children Aged 3–6* promulgated by the Ministry of Education in 2012. By contrast, TGMD, developed by U.S. scholars, is a process-oriented evaluation for children that can test the quality of their movement. The present study proposes both a process-oriented and a results-oriented evaluation method to develop a suitable tool for measuring the development of the FMS of Chinese children.

#### 4.3.2. Functional Setting

Existing scales are mainly screening tools with specific uses. For example, KTK assesses children’s movement ability to screen those with brain damage and behavioral disorders. M-ABC is a screening tool for assessing developmental coordination disorders in children. BOTMP is mainly used to measure movement deficits in children and disability problems in adolescents, such as cerebral palsy, developmental coordination disorder, attention deficit disorder, and autism. By contrast, the proposed scale examines the development of the FMS of all preschool children.

#### 4.3.3. Age Range

Some international scales examine an extensive age range and are thus unsuitable for evaluating preschool children in China (i.e., children aged 3–6 years who have not yet entered primary school). For example, BOTMP applies to children aged 4–14, M-ABC is suitable for ages 4–12, and KTK is suitable for ages 5–14. Hence, the proposed scale can accurately evaluate the FMS development of preschool children.

#### 4.3.4. Test Content

The contents of the test were expanded to include hitting the ball with one hand and catching a bouncing ball. Additionally, hitting a ball with one hand increased the requirement of throwing accuracy compared with the overhand throwing of TGMD-2.

### 4.4. Project Rating

To score the FMS of preschool children, each project must carry out two tests. Scoring for each item consists of 4–5 standards. A score of “1” is awarded for each standard that is met, and a score of “0” for failing to meet the standard. After the evaluation, the scale could provide each test’s original, standard, and total scores. Then, the standard score is converted, consistent with the TGMD-2 test standard.

### 4.5. Adaptability to Chinese Culture

First of all, the actual measurement indicators of Chinese children were selected according to the physical development of Chinese children. In the selection of test indicators, an index system can be established which can reflect the development of children’s movements comprehensively and objectively. At present, most of these evaluation tools are developed with the western population as the norm. Due to the objective existence of cultural and racial differences between the East and the West, it is doubtful whether these evaluation scales can be directly applied to the physical fitness measurement of Asian children.

Secondly, in the development of measurement tools, specific measurement methods should be selected according to different types of movements (large muscle group movements and fine movements). According to the rules of Chinese children’s bone and body development, the model was built based on the data of Chinese children, and the self-evaluation criteria were set.

Thirdly, when evaluating children’s movement development levels, it is necessary to establish diversified and differentiated evaluation criteria by referring to children’s movement development norms at different ages as well as children’s physical constitution, nutritional status, living environment and other factors.

Fourth, based on the comprehensive analysis and summary of relevant literature, the item pool of this scale was established by extracting the basic movements of children, combining with the characteristics of China, and adding the relevant movements that are more in line with the educational background of preschool children in China, such as “racket horizontal hitting fixed table tennis”. Although it was not the final test item, the authors hope that the test content will be with more and more Chinese characteristics.

### 4.6. Limitations

Since this project was approved in March 2020, due to the impact of COVID-19, the collection of research samples originally planned to cover the whole country was restricted. Due to the strict closed management adopted by some kindergartens and early childhood institutions because of the national plan, the test samples were compressed. The subject focuses on developing test indicators for preschool children in Northeast China, and there may be errors when used in other areas. To this end, it is necessary to continue research on the subject step by step and complete the measurement and sampling of children with various demographic characteristics across the country (Children with different regions, including urban and rural areas, physique, nutritional status, living environment, economic characteristics, etc.) to improve the effectiveness of the scale.

## 5. Conclusions

In this study, we built a suitable 12-item scale for measuring the FMS of preschool children in northeast China. The proposed scale comprised three dimensions: physical locomotion skills (five items), object control skills (five items), and physical stability skills (two items). An inter-rater reliability test, a test/retest test, a homogeneity test, and a construct validity test were then conducted on the final measurement scale. The results demonstrated that the implementation of the test content and method standard was relatively uniform among the raters, the measurement method and standard had high stability, and the internal consistency of the measurement content and construct validity were very good. Hence, the measurement scale constructed in this study is verified as an effective tool for measuring the FMS of preschool children in northeast China.

The proposed scale has several practical applications. First, understanding the FMS development level of preschool children can shed light on their physical condition and be used as an essential indicator for evaluating and monitoring their physical development. Therefore, monitoring FMS development is of great significance in early childhood education. Second, measuring and understanding FMS development regularly throughout the growth stage of preschool children can help demonstrate weak aspects in a timely manner and allows for interventions to adjust the educational content of the next stage. Finally, it is necessary to adjust the educational process according to the measurement results, optimize educational efficiency, and comprehensively improve the physical development level of preschool children in this sensitive period of their movement skills development. In the future, based on improving the fundamental movement skills evaluation scale for Chinese children, research should be conducted on promoting motor skills to preschool children’s physique and teaching movement skills to preschool children.

## Figures and Tables

**Table 1:** Participants in developing the preliminary and final measurement scales.

Participants		Gender	*n*	Age in Months	Height (m)	Weight (kg)	BMI (kg/m^2^)
				(M ± SD)			
The preliminary measurement scale	36–47 months	M	10	44.50 ± 2.92	1.00 ± 0.03	15.90 ± 1.43	15.85 ± 0.82
		F	10	42.50 ± 3.24	0.99 ± 0.37	15.37 ± 1.55	15.48 ± 1.23
	48–59 months	M	10	54.30 ± 2.87	1.07 ± 0.05	18.80 ± 2.20	16.52 ± 1.31
		F	10	55.30 ± 3.71	1.07 ± 0.04	19.37 ± 2.97	16.78 ± 1.60
	60–72 months	M	10	66.60 ± 2.91	1.10 ± 0.05	22.25 ± 8.60	18.18 ± 4.93
		F	10	67.50 ± 3.24	1.12 ± 0.07	22.01 ± 4.31	17.55 ± 1.65
The final measurement scale	36–47 months	M	40	42.95 ± 4.03	0.99 ± 0.04	16.64 ± 1.63	17.12 ± 1.20
		F	40	42.20 ± 3.07	0.97 ± 0.03	15.46 ± 1.40	15.30 ± 4.64
	48–59 months	M	75	53.45 ± 2.92	1.06 ± 0.05	19.66 ± 3.02	17.52 ± 1.81
		F	57	53.52 ± 4.04	1.03 ± 0.05	17.84 ± 2.80	16.63 ± 1.74
	60–72 months	M	43	63.75 ± 3.86	1.10 ± 0.06	21.05 ± 5.61	17.12 ± 3.21
		F	43	64.78 ± 4.18	1.10 ± 0.06	20.63 ± 3.67	17.10 ± 1.87

Abbreviations: M: mean; SD: standard deviation; BMI: body mass index; M: boys, F: girls.

**Table 2:** Commonly used children’s movement skill measurement scales globally.

No.	Country	Year of Publication	Author(s)	Name
1	Germany	1970	Kiphard and Schilling	Körperkoordinationstest für Kinder (KTK) [60]
2	United States	1974	Folio and Fewell	Peabody Developmental Motor Scales [61]
3	Canada	1978	R.H. Bruininks and B.D. Bruininks	Bruininks-Oseretsky Test of Motor Proficiency (BOTMP) [62]
4	United States	1985	Dale A. Ulrich	Test of Gross Motor Development (TGMD) [63]
5	Germany	1987	Zimmer and Volkamer	Motoriktest für vier-bis sechsjährige Kinder [60]
6	England	1992	Henderson and Sugden	Movement Assessment Battery for Children (M-ABC) [64]
7	Netherlands	2004	Vles, Kroes, and Feron	Maastrichtse Motoriek Test (MMT) [65]
8	China	2010	Sun et al.	Preschooler Gross Motor Quality Scale [66]
9	Canada	2015	Longmuir et al.	Canadian Agility and Movement Skill Assessment [67]
10	China	1992	Rong and Houcan	Child Development Scale for 3–6-Year-Olds [68]
11	China	2000	State General Administration of Sport research team	National Physical Fitness Standards (for Young Children) [56]

**Table 3:** The results of the expert questionnaire.

	Kendall’s W^a^	*p*
Physical locomotion skills	0.350	0.000 ***
Object control skills	0.223	0.000 ***
Physical stability skills	0.276	0.000 ***

*** *p* < 0.01.

**Table 4:** Results of the questionnaire items.

Item		Average Score (M ± SD)	CoV	Cumulative Percentage (%)
Physical locomotion skills	Straight line running	4.81 ± 0.51	0.11	90.48%
	Shuttle running	4.71 ± 0.56	0.12	95.24%
	Changeover running	4.24 ± 1.09	0.26	80.95%
	One-foot jump	4.43 ± 1.08	0.24	90.48%
	Standing long jump	4.48 ± 0.68	0.15	90.48%
	Front slide	3.57 ± 0.98	0.27	52.38%
	Sidestep slide	4.05 ± 0.80	0.20	76.19%
	Running jump	3.43 ± 1.08	0.31	52.38%
	Jumping continuously with both feet sideways	3.71 ± 1.06	0.28	61.90%
	Stride jump	4.19 ± 0.93	0.22	76.19%
	Jumping jacks	3.76 ± 1.18	0.31	61.90%
	Synchronized jump on the same side	3.10 ± 1.09	0.35	38.10%
	Jumping obstacle	3.95 ± 0.74	0.19	71.43%
Object control skills	Bouncing a ball in place	4.57 ± 0.87	0.19	85.71%
	Catching a ball with both hands	4.67 ± 0.73	0.16	95.24%
	Kicking a ball	4.57 ± 0.60	0.13	95.24%
	Throw a ball up with both hands	4.62 ± 0.67	0.14	90.48%
	Hitting a ball with one hand over the shoulder	4.14 ± 0.91	0.22	85.71%
	Bocce ball	4.05 ± 1.12	0.28	71.43%
	Overhand pitching	3.90 ± 0.89	0.23	66.67%
	Catching a bouncing ball	4.24 ± 0.89	0.21	80.95%
	Holding a ball and walking	3.71 ± 1.19	0.32	57.14%
	Racket horizontal hitting fixed table tennis	3.62 ± 1.40	0.39	57.14%
Physical stability skills	Standing on one foot	4.76 ± 0.70	0.15	95.24%
	Hanging on a horizontal bar	3.48 ± 1.50	0.43	47.62%
	Walking on a balance beam	4.43 ± 0.93	0.21	80.95%
	Straight walking	3.95 ± 1.02	0.26	66.67%
	Standing back and forth on two feet	3.95 ± 1.24	0.31	66.67%
	Squatting	4.05 ± 1.36	0.34	71.43%
	Backward straight walking	4.00 ± 1.05	0.26	76.19%
	Forward rolling	3.14 ± 1.01	0.32	33.33%
	Tiptoe walking	4.29 ± 1.10	0.26	80.95%
Abbreviations: M: mean; SD: standard deviation				*n* = 21

**Table 5:** Results of testing the preliminary measurement scale.

Item		Average Score (M ± SD)	CoV	rho
Physical locomotion skills	Straight line running	9.15 ± 2.98	0.33	0.498 **
	Shuttle running	8.38 ± 1.23	0.15	0.465 **
	Stride jump	6.82 ± 2.09	0.31	0.565 **
	One-foot jump	7.59 ± 1.91	0.25	0.645 **
	Sidestep slide	8.62 ± 2.27	0.26	0.618 **
	Standing long jump	7.00 ± 2.39	0.34	0.421 **
	Physical locomotion total score	46.42 ± 1.27	0.03	
Object control skills	Bouncing a ball in place	5.02 ± 6.24	1.24	0.694 **
	Catching a ball with both hands	6.54 ± 2.60	0.40	0.478 **
	Kicking a ball	7.75 ± 1.34	0.17	0.731 **
	Hitting a ball with one hand	7.69 ± 2.09	0.27	0.750 **
	Catching a bouncing ball	8.36 ± 2.03	0.24	0.637 **
	Object control total score	35.36 ± 1.96	0.06	
Physical stability skills	Standing on one foot	7.29 ± 6.54	0.90	0.600 **
	Walking on a balance beam	5.71 ± 1.13	0.20	0.778 **
	Balance total score	13.00 ± 1.74	0.13	
	Total score	94.78 ± 2.17	0.02	
rho: Spearman’s rank correlation rho; Abbreviations: M: mean; SD: standard deviation; ** *p* < 0.01				*n* = 60

**Table 6:** Results of testing the final measurement scale.

Item	Inter-Rater Reliability (*n* = 298)		Test/Retest Reliability (*n* = 36)		Homogeneity (*n* = 298)
	Spearman	Sig.	Spearman	Sig.	Cronbach’s α
Straight line running	0.929 **	0.000	0.913 **	0.000	0.846
Stride jump	0.951 **	0.000	0.926 **	0.000	0.841
One-foot jump	0.965 **	0.000	0.909 **	0.000	0.840
Sidestep slide	0.975 **	0.000	0.914 **	0.000	0.839
Standing long jump	0.914 **	0.000	0.855 **	0.000	0.845
Bouncing a ball in place	0.959 **	0.000	0.928 **	0.000	0.839
Catching a ball with both hands	0.937 **	0.000	0.772 **	0.000	0.846
Kicking a ball	0.966 **	0.000	0.936 **	0.000	0.844
Hitting a ball with one hand	0.974 **	0.000	0.956 **	0.000	0.841
Catching a bouncing ball	0.970 **	0.000	0.914 **	0.000	0.843
Standing on one foot	0.965 **	0.000	0.835 **	0.000	0.845
Walking on a balance beam	0.844 **	0.000	0.900 **	0.000	0.844

** Significance at the 1% level (two-tailed).

## Data Availability

Data provided in this study are available upon request by the corresponding author. The data were not made public because they involved the personal privacy issues of the participants.
